# Sign of Retroperitoneal Rupture of Colon

**Published:** 1916-10

**Authors:** 


**Sign of Retroperitoneal Rupture of Colon.** Andre Chalier, Rev. de Chir., Aug. 1914-Nov. 1915, noted emphysema of the scrotum on the same side as a penetrating shrapnel wound, within 24 hours, the intestinal gases following the spermatic vessels. Recovery after operation.

